# Concentration of Zinc, Copper, Selenium, Manganese, and Cu/Zn Ratio in Hair of Children and Adolescents with Myopia

**DOI:** 10.1155/2019/5643848

**Published:** 2019-04-10

**Authors:** Monika Fedor, Beata Urban, Katarzyna Socha, Jolanta Soroczyńska, Małgorzata Krętowska, Maria Halina Borawska, Alina Bakunowicz-Łazarczyk

**Affiliations:** ^1^Department of Pediatric Ophthalmology and Strabismus, The Medical University of Bialystok Children's Clinical Hospital, Waszyngtona 17, 15–274 Bialystok, Poland; ^2^Department of Pediatric Ophthalmology and Strabismus, Medical University of Bialystok, Waszyngtona 17, 15-274 Bialystok, Poland; ^3^Department of Bromatology, Medical University of Bialystok, Mickiewicza 2D, 15–222 Białystok, Poland; ^4^Faculty of Computer Science, Bialystok University of Technology, Wiejska 45A, 15-351 Białystok, Poland

## Abstract

The aim of this work was to determine the concentration of trace elements, zinc, copper, selenium, manganese, and Cu/Zn ratio, in scalp hair samples of children and adolescents with myopia. The study included 92 children (mean age 14.5 ± 2.5 years) with myopia and 43 healthy persons (mean age 11.8 ± 4.7 years). Each patient had a complete eye examination. Trace element concentrations in hair were determined by atomic absorption spectrometry. Cu/Zn ratio was also calculated. The zinc level in the hair of myopic patients was significantly higher (260 *µ*g/g) in comparison to the control group (130 *µ*g/g). There was a significantly lower Cu/Zn ratio in myopic patients (0.045) compared with controls (0.07). An insignificant difference was observed in the hair level of copper, selenium, and manganese between patients and controls. The results show that trace elements may play a significant role in the pathogenesis of myopia. Further studies should pay more attention to determine the effect of trace element on children myopia.

## 1. Introduction

Myopia is an important health and social issue in the world today. It is estimated that, in the year 2020, one-third of the world population will be suffering from myopia. It is especially worrying that an increasing number of myopia cases are observed among children. High myopia (≥6 D) may lead to vision loss as a consequence of, e.g., retinal detachment, myopic choroidal neovascularization, glaucoma, or cataract [1]. The etiology of myopia is complex and not fully researched—genetic and environmental factors are mentioned [2]. Among others, the influence of oxidative stress on the development of myopia is emphasized [3]. Zinc (Zn), cooper (Cu), manganese (Mn), and selenium (Se) play a very important role in the antioxidative processes [4–9] and in the rebuilding of the sclera [10–13]; therefore, we have chosen these trace elements for our study.

Despite a substantial amount of biological material (blood, blood serum, urine, nails, and hair) in which trace elements can be determined, hair has some advantage over the others. Determining microelements in blood or urine samples may not reflect the correct amount of microelements in the entire organism due to homeostatic mechanisms which balance out potential insufficiencies [14–20]. Hair samples are painless to collect, easy to store and transport, and can be easily purified from environmental pollutants. Additionally, the amount of microelements contained in hair samples reflects the mineral status of the organism from months before the examination [18–24].

Research is being made on using hair as a biological material in diagnosing many diseases, such as Parkinson's disease, arterial hypertension, schizophrenia, Down's syndrome, and autism [18–21, 25–27]. Unfortunately, only one research has been carried out for the determination of trace elements in the hair of patients with myopia [28].

The purpose of this study was to estimate Zn, Cu, Mn, and Se concentration and Cu/Zn ratio in the hair of children and adolescents with moderate and high myopia.

## 2. Materials and Methods

In our previous paper from 2017, we have presented the serum concentration of Zn, Cu, Mn, and Se in children and adolescents with myopia [29]. The concentration of microelements was determined by the atomic absorption spectrometry method (ASA), flameless technique with electrothermal atomization in a graphite tube (Cu, Se, and Mn), and atomization in acetylene-air flame (Zn) with the Zeeman background correction (Z-2000 instrument, Hitachi, Japan). In the current study, we have decided to analyse the content of these microelements in the hair of young myopic subjects, using atomic absorption spectrometry.

### 2.1. Participants

The characteristics of control and myopic patients groups are presented in Table 1.

Ninety-two patients (62 girls, 30 boys) aged 7–17 years with myopia (mean −7.03 ± 2.47 D) were selected for the present study. Myopia was defined as spherical equivalent in both eyes ≤−0.5 D. The patient's mean age was 14.5 ± 2.5 years. Of all myopic patients, we separated 71 individuals with the same bilateral grade of myopia: 48 patients with high myopia (HM) in both eyes (spherical equivalent < −6.0 D, mean −8.29 ± 2.17 D; and 23 patients with moderate myopia (MM) in both eyes (spherical equivalent from −3.0 D to −6.0 D, mean −5.28 ± 0.51 D. The other 21 patients had HM in one eye and MM in the second one. All these myopic patients were hospitalized in the Department of Pediatric Ophthalmology and Strabismus, Medical University of Bialystok, Poland. Exclusion criteria were any systemic disease, history of any anterior or posterior eye segment disease, following the diet, the mineral supplements consumption in the last 6 months, and tinted hair. The control group consisted of 43 healthy individuals (24 girls, 19 boys, with a mean age of 11.8 ± 4.7 years), who had spherical equivalent from −0.5 D to +0.5 D.

A complete ophthalmologic examination was performed in all persons, who were enrolled in the study. Concentration of trace elements (Zn, Cu, Se, and Mn) and Cu/Zn ratio in hair samples were determined in children and adolescents with myopia and in the control group.

The study was approved by the Local Ethical Committee (R-I-002/402/2016), and written consent was taken from the parents or statutory representants of the patients. They were informed about the study, and their participation was voluntary.

### 2.2. Determination of Microelements and Cu/Zn Ratio

Hair samples (3-4 cm from the scalp) were taken from several places from the occipital part. The hair Zn, Cu, Se, and Mn levels and Cu/Zn ratio were determined in the Department of Bromatology, Medical University of Bialystok. Hair samples were washed with acetone, deionized water, and absolute ethanol, and then dried at 80°C for 24 hours. About 0.2 g of the sample was weighed with accuracy to 1 mg and wet mineralized in concentrated nitric acid (HNO_3_) in a microwave closed system (Berghof, Germany). The concentrations of microelements were determined by atomic absorption spectrometry, with Zeemans background correction (Hitachi Z-2000, Japan), with acetylene-air flame (in the case of Cu and Zn) and electrothermal atomization in a graphite tube (in the case of Mn and Se). The accuracy control of the methods used was made on a certified reference material—Human Hair GBW 09101 (China). Validation parameters, such as accuracy, precision, and detection limit, were estimated (Table 2).

Also, the content of Cu and Zn was used to calculate Cu/Zn ratio.

### 2.3. Statistical Analysis

The analysis was conducted with the use of STATISTICA version 13.1 and PRISM version 5.01. Because the hypothesis of normal distribution of the analysed parameters was rejected (Shapiro–Wilk test), the nonparametric tests were applied. The Mann–Whitney *U* test was used to compare the two groups of patients while Kruskal–Wallis test with Dunn's multiple comparison test, to compare the parameters among control, HM, and MM groups. Additionally, the Spearman's rank correlation coefficient was used as a correlation measure between variables. The data were characterized by a minimum, median, and maximum value. To compare the median values of serum trace elements concentration and Cu/Zn ratio using the Mann–Whitney *U* test and the Kruskal–Wallis test; the Bonferroni adjusted significance level of 0.01 was applied. For other tests, we used the significance level of 0.05.

## 3. Results

Table 3 shows the concentration of Zn, Cu, Se, and Mn and Cu/Zn ratio in the hair of patients with myopia and in the control group.

In the case of Zn, mean values were significantly higher (*p* < 0.001) in patients with myopia (260 *µ*g/g) than controls (130 *µ*g/g).

The level of Cu was 10.7 *µ*g/g in the hair of myopic patients and 8.91 *µ*g/g in the hair of the healthy controls, and these differences were not significant. We observed lower (*p* < 0.001) Cu/Zn ratio in the study group in comparison to the control group (0.045 and 0.07, respectively).

The next examined element was Se. Its median concentration in the hair of myopic patients was 337 ng/g and 416 ng/g in the hair of the control group, and these differences were not significant.

The values for Mn in the hair of patients with myopia were 459 ng/g and it was not different from the mean Mn concentration the in hair of the control group (297 ng/g).

**Figure 1:**
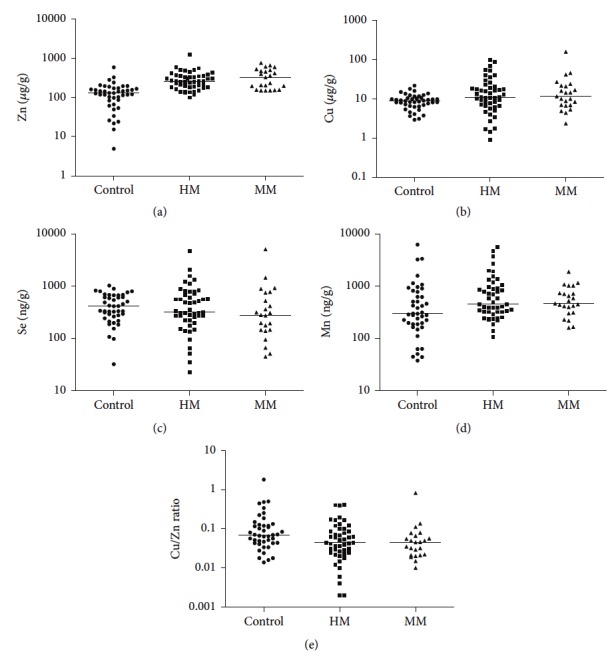
Hair trace elements (Zn, Cu, Se, and Mn) concentration and Cu/Zn ratio in the control group compared to patients with MM group and HM group.

The concentrations of Zn, Cu, Se, and Mn and Cu/Zn ratio in the hair of patients with moderate myopia (MM) and high myopia (HM) are presented in Table 4 and Figure 1.

We observed higher content of Zn in the hair of patients with moderate myopia and high myopia in comparison to the control group (*p* < 0.001). There were no differences in Zn hair concentration between MM and HM groups (Table 4, Figure 1).

There were no differences in Cu, Se, and Mn concentration and Cu/Zn ratio between MM and HM groups and the healthy controls (Table 4, Figure 1).

**Figure 2:**
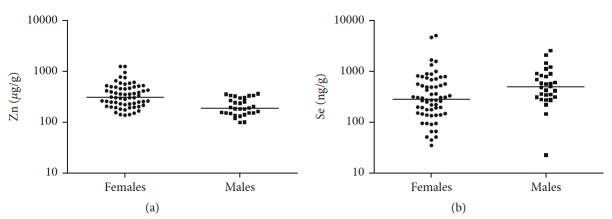
Zinc and selenium concentration in the hair samples of myopic patients depending on gender.

Table 5 and Figure 2 report trace elements levels in the examined patients depending on sex.

When comparing females and males, the mean concentration of Zn was higher in females than in males in myopic patients (Figure 2). There was a significantly lower hair Se concentration in myopic females in comparison with myopic males (Figure 2). Regarding other microelements, there were no statistically significant differences in the control group and the myopic group depending on gender.

Correlations between hair trace elements concentration in the control group and in myopic patients are presented in Tables 6 and 7.

There was a negative correlation between Zn concentration and Cu/Zn ratio in the control and study group. We observed positive correlation between Cu level and Cu/Zn ratio in the study and control group (Tables 6 and 7). Zn correlated negatively with Se in myopic patients (*r* = −0.31, Table 7). It was found that only the concentration of Cu in myopic patients had a significant positive correlation with age (*r* = 0.21, Table 7).

## 4. Discussion

The amount of trace elements in the human body is relevant to the number of elements supplied externally, the body's demand, and their elimination [20]. Limited data are available on the relation between the level of trace elements in hair and myopia. We have quantified Zn, Cu, Mn, and Se because their role in the myopia pathogenesis is speculated.

Zn plays an important role in the organism. It shows antioxidative activity and is also a component of numerous enzymes and proteins [20, 30]. Zn reduces the activities of oxidant-promoting enzymes and inhibits lipid peroxidation products. The lack of Zn increases oxidative stress and enhances oxidative damage to DNA, proteins, and lipids [3].

The pathologic changes of myopic sclera include disruption of the packing of fibrils and fibres, a decrease in the average diameter of the cross section of fibrils, an increased fraction of soluble collagen, a reduction in transverse cross-linking of the scleral collagen, and a decrease in its resistance to proteolytic enzyme [31]. The elongation of the eyeball in the course of myopia is related to the biochemical remodeling of the sclera, which is composed of collagen fibrils, proteoglycans, and glycoproteins [31, 32]. Zinc is involved in collagen metabolism, it builds the active center of matrix metalloproteinases, which take place in collagen remodeling, and it reduces the activity of lysyl oxidase, which participates in collagen synthesis [33]. Huibi et al. in their experimental study observed the inhibition of elongation of the chicks eyeballs, when they were supplemented with zinc [6]. They concluded that zinc can be used to prevent and treat myopia to a certain extent. Improper zinc levels were registered in blood serum, hair, and tears of patients with myopia [30]. Homeostatic mechanisms quickly balance out zinc insufficiency in blood; therefore, the determined levels of Zn in blood serum can differ from the amount of the element in hair samples [29]. Cai did research on the levels of zinc and copper in the hair of 20–24 years old students in Jinzhou, China [28]. He observed that zinc levels in hair of students with myopia were lower than their healthy peers. Additionally, he noticed a decreased level of this element in hair samples proportionally to the increase of myopia. He speculated that myopic degree may be related with the lack of some trace elements. Our research showed contrasting results (Table 3). The zinc level in the hair of patients with myopia was significantly greater than in the control group. Moreover, the Zn level median in the hair of myopic girls was 1.5 times higher than that in the hair of boys with myopia (Table 5). Interestingly, our previous research on a similar group of children showed lower Zn level in blood serum of children with myopia [29]. We can assume that in patients with myopia occurs an accumulation of Zn in their hair, with a resulting insufficiency of this element in the blood and thus its lesser availability for the eyes. This occurrence is yet to be confirmed in further research. Despite the fact that the overdose of zinc is very rarely observed, in the present situation, it is best to supplement this microelement carefully and individually, checking its level both in the blood and in the hair.

Cu is an important catalyst of enzymes partaking in oxidoreductions of, inter alia, lysyl oxidase and Cu/Zn SOD superoxide dismutase [7, 10–12]. Lysyl oxidase takes part in the spontaneous formation of chain cross links in scleral collagen, regulates fibrillogenesis, and is essential for correct collagen fibril shape formation [33, 34]. Normal Cu metabolism is essential to ocular tissue and is associated with myopic refractive error development [28]. In an experimental study, sub-Tenon's capsule injection of Cu compounds resulted in increased scleral Cu concentration, activation of collagen biosynthesis processes, and improved scleral tissue elasticity [12]. In the current study, we did not observe significant differences in Cu levels in the hair of patients with myopia when compared to the control group (Table 3). On the contrary, students with myopia in the research of Cai had higher levels of Cu in the hair and there was a positive correlation between the Cu level and refractive error [28]. Due to higher levels of Zn in the hair of myopic children, surprisingly, Cu/Zn ratio was significantly smaller in the myopic group of our research. Cu/Zn ratio has been proven of diagnostic value in a number of human disorders, including pediatric infectious diseases, such as tuberculosis [35]. The diagnostic value of the Cu/Zn ratio as a disease marker was also shown in hypertension, autism, and neoplastic diseases [36–38]. Cu/Zn ratio is a good indicator of oxidative stress in serum, but, in our opinion, it seems that in the hair, it is not as useful indicator of oxidative stress as it seemed initially, maybe because the organ of vision is in some way “isolated”.

Se acts as an essential cofactor required to activate several enzyme systems in the human body. It also displays antioxidative properties. It is a component of the glutathione peroxidase, which disables the oxygen-free radicals [19, 29]. Therefore, selenium could be efficacious in preventing disorders attributed to oxidative stress. The Se level in the present research did not differ significantly between the control group and the examined group. The observed levels spanned noticeably. Among the myopic patients, the median Se level was 1.5 times higher in boys than in girls.

Mn is a trace element essential for the synthesis of collagen, it also builds manganese-dependent superoxide dismutase (MnSOD) [8, 30]. MnSOD is the most significant antioxidant enzyme in protecting against reactive oxygen species in the human body. Regarding Mn level, we did not observe statistically significant differences between the two groups. Similarly, in our previous study, we did not observe differences in serum concentration of manganese in the study and the control group [29]. Both Se and Mn concentrations in the hair of myopic subjects were examined, to our knowledge, for the first time, so more extensive research is necessary to validate their role in the pathogenesis in myopia.

The current study has some limitations, one being the small sample size. Another limitation was the lack of randomization. Additionally, we have no nutritional assessment and no information about environmental and occupational exposures; therefore, further research is needed on the role of trace elements in myopia.

## 5. Conclusions

Although the idea of measuring trace elements in young myopic subjects' hair is tempting, the present study shows that only Zn hair level was higher in comparison to the control group. At the same time, in the studied patients, there were no significant differences in hair concentration of Cu, Se, and Mn in comparison to the control group. From these results, we concluded that excess zinc in the hair of children and adolescents with myopia may be associated with the development and progression of myopia; however, further studies are needed to determine the effect of trace elements on children myopia.

## Figures and Tables

**Table 1:** Characteristics of control and myopic patients groups.

	Control group	Myopic patients	*P*
Age			
Mean ± std (range)	11.8 ± 4.7 (4, 17)	14.5 ± 2.5 (7, 17)	0.011^*∗*a^
Min, med, max	4, 12, 17	7, 15, 17	

Sex			
Female/male	24/19	62/30	0.19^*∗*b^

^a^ *P* value for Mann–Whitney *U* test. ^b^ *P* value for a test for two proportions. ^*∗*^Statistically significant differences at 0.05 significance level.

**Table 2:** Validation parameters of the methods used for the determination of microelements.

Microelement/method	Dilution factor	Recovery of the CRM (%)	Detection limit	Accuracy (%)	Precision (coefficient of variation) (%)
Cu/FAAS	25	95	0.158 ppm	2.2	5.1
Zn/FAAS	250	101	0.013 ppm	1.3	2.1
Mn/ET AAS	125	98	0.084 ppb	1.2	2.1
Se/ETAAS	25	99	1.74 ppb	0.4	1.9

FAAS: flame atomic absorption spectrometry; ETAAS: electrothermal atomic absorption spectrometry; ppm: *µ*g/g; ppb: ng/g.

**Table 3:** Trace elements concentration and Cu/Zn ratio in the hair of the control group and the myopic patients.

Element	Control group (*n*=43), min; med; max	Myopic patients (*n*=92), min; med; max	*P* ^a^
Zn (*µ*g/g)	4.91; 130; 588	98; 260; 1240	<0.001^*∗*^
Cu (*µ*g/g)	2.95; 8.91; 21.9	0.34; 10.7; 664	0.049
Se (ng/g)	32.2; 416; 1030	22.6; 337; 5060	0.58
Mn (ng/g)	37.8; 297; 6240	107; 459; 5610	0.013
Cu/Zn ratio	0.014; 0.07; 1.82	0.001; 0.045; 0.82	0.001^*∗*^

^a^ *P* value for Mann–Whitney *U* test. ^*∗*^Statistically significant differences at 0.01 significance level.

**Table 4:** Hair trace elements concentration and Cu/Zn ratio in HM and MM groups.

	HM group (*n*=48), min; med; max	MM group (*n*=23), min; med; max	*P* ^a^	Significant differences
Zn (*µ*g/g)	99.6; 258; 1240	149; 322; 754	<0.001^*∗*^	C vs HM, C vs MM
Cu (*µ*g/g)	0.89; 10.9; 98.1	2.34; 11.5; 158	0.04	—
Se (ng/g)	22.6; 323; 4670	44.6; 280; 5060	0.42	—
Mn (ng/g)	107; 451; 5610	160; 464; 1880	0.037	—
Cu/Zn ratio	0.002; 0.045; 0.41	0.01; 0.045; 0.817	0.017	—

^a^ *P* value for Kruskal–Wallis test. ^*∗*^Statistically significant differences at 0.01 significance level.

**Table 5:** The influence of sex for serum trace elements concentration and Cu/Zn ratio in control and myopic patients groups.

	Min; med; max	Min; med; max	*P* ^a^
*Control group*	*Females (n*=24)	*Males (n*=19)	
Zn (*µ*g/g)	4.91; 136; 326	15.3; 118; 588	0.78
Cu (*µ*g/g)	3.1; 8.28; 18	2.95; 9.49; 21.9	0.63
Se (ng/g)	32.2; 334; 1030	155; 487; 824	0.28
Mn (ng/g)	37.8; 299; 3360	44.9; 281; 6240	0.44
Cu/Zn ratio	0.02; 0.07; 1.82	0.01; 0.07; 0.5	0.82

*Myopic patients*	*Females (n*=62)	*Males (n*=30)	
Zn (*µ*g/g)	137; 307; 1240	98; 186; 361	<0.001^*∗*^
Cu (*µ*g/g)	0.34; 13.2; 664	1.72; 9.12; 158	0.2
Se (ng/g)	34.7; 285; 5060	22.6; 495; 2550	0.008^*∗*^
Mn (ng/g)	107; 471; 4710	150; 402; 5610	0.22
Cu/Zn ratio	0.01; 0.04; 0.70	0.01; 0.05; 0.82	0.36

^a^ *P* value for Mann–Whitney *U* test. ^*∗*^Statistically significant differences at 0.01 significance level.

**Table 6:** Spearman's rank correlation coefficients for control group.

	Zn	Cu	Se	Mn	Cu/Zn ratio
Age	0.01	−0.08	0.03	−0.01	0.04
Zn	1.00	−0.08	0.06	0.23	−0.82^*∗*^
Cu	—	1.00	−0.18	0.10	0.58^*∗*^
Se	—	—	1.00	0.06	−0.14
Mn	–	—	—	1.00	−0.19
Cu/Zn ratio	—	—	—	—	1.00

^*∗*^Statistically significant correlation at 0.05 significance level.

**Table 7:** Spearman's rank correlation coefficients for myopic patients.

	Zn	Cu	Se	Mn	Cu/Zn ratio
Age	0.10	0.21^*∗*^	−0.09	−0.09	0.11
Zn	1.00	0.15	−0.31^*∗*^	0.09	−0.34^*∗*^
Cu	—	1.00	−0.04	−0.06	0.85^*∗*^
Se	—	—	1.00	0.03	0.15
Mn	—	—	—	1.00	−0.06
Cu/Zn ratio	—	—	—	—	1.00

^*∗*^Statistically significant correlation at 0.05 significance level.

## Data Availability

The data used to support the findings of this study are available from the corresponding author upon request.
